# Improved PNIPAAm-Hydrogel Photopatterning by Process Optimisation with Respect to UV Light Sources and Oxygen Content

**DOI:** 10.3390/gels2010010

**Published:** 2016-03-04

**Authors:** Sebastian Haefner, Mathias Rohn, Philipp Frank, Georgi Paschew, Martin Elstner, Andreas Richter

**Affiliations:** 1 Polymeric Microsystems, Institute of Semiconductors and Microsystems, Technische Universität Dresden, 01062 Dresden, Germany; sebastian.haefner@tu-dresden.de (S.H.); philipp.frank@tu-dresden.de (P.F.); georgi.paschew@tu-dresden.de (G.P.); 2 Physical Chemistry of Polymers, Department of Chemistry and Food Chemistry, Technische Universität Dresden, 01062 Dresden, Germany; mathias.rohn@tu-dresden.de; 3 Center for Advancing Electronics Dresden (cfaed), Technische Universität Dresden, 01062 Dresden, Germany; martin.elstner@tu-dresden.de

**Keywords:** photopatterning, photopolymerisation, PNIPAAm, stimuli-responsive hydrogel, reproducibility

## Abstract

Poly-*N*-isopropylacrylamide (PNIPAAm) hydrogels, known for their sensor and actuator capabilities, can be photolithographically structured for microsystem applications. For usage in microsystems, the preparation, and hence the characteristics, of these hydrogels (e.g., degree of swelling, size, cooperative diffusion coefficient) are key features, and have to be as reproducible as possible. A common method of hydrogel fabrication is free radical polymerisation using a thermally-initiated system or a photoinitiator system. Due to the reaction quenching by oxygen, the polymer solution has to be rinsed with protective inert gases like nitrogen or argon before the polymerisation process. In this paper, we focus on the preparation reproducibility of PNIPAAm hydrogels under different conditions, and investigate the influence of oxygen and the UV light source during the photopolymerisation process. The flushing of the polymer solution with inert gas is not sufficient for photostructuring approaches, so a glove box preparation resulting in better quality. Moreover, the usage of a wide-band UV light source yields higher reproducibility to the photostructuring process compared to a narrow-band UV source.

## 1. Introduction

Hydrogels are 3D polymer networks with the capability to swell in a swelling agent (e.g., water [1]). During the swelling process, the gel can take up multiples of its dry mass of water [2]. This type of polymer can be chemically tuned regarding its chemical, physical, or mechanical properties [3]. By functionalisation, a polymeric network can act as storage containers for enzymes, antibodies, or DNA [4,5]. By changing the cross-linker concentrations, the elastic modulus [6] of the hydrogel can be altered from very soft to rubber-like so that an adjustment of mechanical requirements becomes possible. Hydrogel copolymers produced by mixing different monomers in diverse ratios also affects the hydrogel properties. So far, a variety of hydrogel compositions incorporating various functional properties have been published and commercialised [7]. Promising features have been presented for the fields of drug delivery systems [8,9], as 3D scaffolds for tissue engineering [10,11,12], and as an active material in microfluidic valves [13] and chemostats [14,15].

Popular hydrogels for microsystem engineering approaches are PNIPAAm (Poly-*N*-isopropylacrylamide)-based hydrogels [16,17]. Such smart hydrogels react to environmental condition changes with an altered degree of swelling [18,19,20]. PNIPAAm hydrogels or derived copolymers are used as microfluidic valves or active material in chemofluidic transistors, and as active components in microfluidic circuits [21]. The ability to pattern these gels by lithography makes PNIPAAm hydrogels applicable for large scale fabrication and high-integration techniques [22]. Therefore, reproducibility of its fabrication process is a key factor for system reliability.

A common method to produce polymers in general, including hydrogels is free radical polymerisation, because of its convenient and time-effective synthesis procedure. A drawback of this approach is the sensitivity to radical quenchers like oxygen. These are strongly reduced or even eliminated by degassing and rinsing of the system with protective gas. However, during the process of photolithographical patterning, the influence of oxygen can hardly be avoided. Most sources of error are related to short exposures to non-oxygen-free atmosphere, to oxygen molecules attached to a material surface, or to diffusion through a tube or seal. In some cases, there is a material which contains oxygen in its volume which slowly diffuse to its surface.In some cases the contamination results from oxygen encapsulated in a material slowly surfacing out. In general, a reaction chamber is filled with prepolymer solution and is selectively exposed to uvUV light through a photomask. We observetested three methods of preparation and concentrate on two of them. The filling and closing of the chamber is a key point of how good oxygen can be avoided:The assembling (filling and closing) of the reaction chamber is most critical in the handling due to a possible contamination with oxygen: 1) A reaction chamber is filled with a polymer solution in a standard air environment and is immediately closed. 2) A tube system is used for rinsing with protective gas and filling with a prepolymer solution. 3) The chamber is filled in a glove-box under an atmosphere with less than 5 ppm oxygen. As preliminary experiments only showed significant difference between method 1) and 3), we concentrate on them. In this paper we focus on the hydrogel preparation conditions for photostructuring by a free radical polymerisation reaction. We show results for different UV light sources (continuous wide-band and pulsed narrow-band), for different exposure times and compare strictly inert and atmospheric handling procedures. The cooperative diffusion coefficients and the degree of swelling were measured as characteristics to get insights onget insights into the reproducibility of the polymerisation process and therefore the hydrogel preparation.

## 2. Results and Discussion

### 2.1. Oxygen and UV Source Influence on the Polymerisation Process

**Figure 1:**
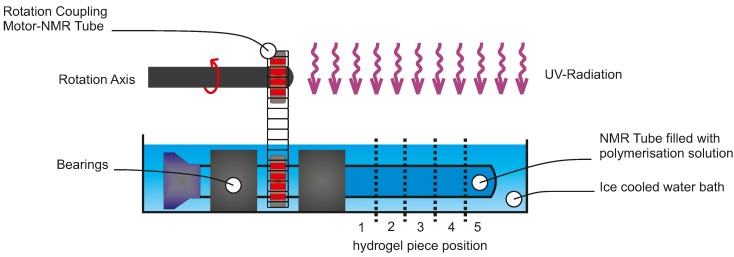
Diagram of the rotation setup for rotating a NMR tube filled with polymerisation solution.

The photopolymerisation homogeneity of PNIPAAm hydrogels was investigated regarding oxygen content after polymerisation setup assembling and UV light source. NMR tubes were filled with polymerisation solution under inert or standard conditions. After sealing, the rotation setup was assembled following the sketch in Figure 1. The NMR tube’s orientation was orthogonal to the UV light radiation. A water bath (0 °C) was used for cooling the solution during the exposure time. Due to the thickness of the tube (outer diameter 5 mm, inner diameter 4.2 mm, length 178 mm), a rotation setup is necessary to avoid a gradient in the gel polymerisation.

**Figure 2:**
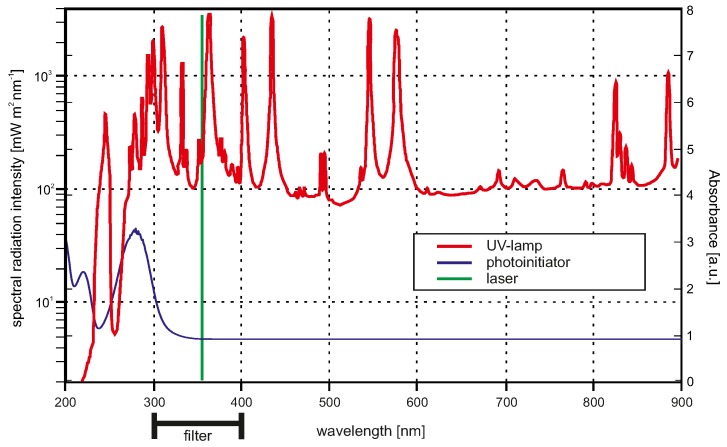
Plot of the spectral radiation intensity at 0.5 m proximity of the mercury-vapour lamp is demonstrated including the UV/Vis spectraspectrum of the photoinitiator Irgacure^®^. The green line sketches the wavelength of the laser with no respect to one of the y-axes. The lamp spectra was reprinted from the lamp datasheet with kindly permission from LOT-Oriel Group Europe.

For UV light radiation we used two different sources: one wide-band and one narrow-band source. A common wide-band source for UV light are mercury-vapour lamps (see Figure 2). A bandpass filter suppresses all wavelengths below 300 nm and all wavelengths above 400 nm, so that the effective bandwidth is between 300 nm to 400 nm. The UV/Vis absorption spectra of the photoinitiator Irgacure^®^ is also plotted in Figure 2. The absorption maximum of the photoinitiator is below 300 nm, and therefore both UV light sources do not fit well to the absorption spectra of the photoinitiator. Nevertheless, for applications in which for instance enzymes should be incorporated in the hydrogels during the polymerisation process, the excitation wavelength should be as high as possible to avoid enzyme damage. Due to safety considerations, moderate UV wavelengths are also preferred.

For the hydrogel polymerisation process we established three parameters, which will be discussed in this section. These parameters are the oxygen content, the exposure time, and the homogeneity of the intensity distribution of the UV light sources. First, the dependency on oxygen content and exposure time is discussed. For each exposure time experiment, a NMR tube was filled with polymerisation solution, coupled in the rotation setup (Figure 1), and exposed to UV light. Afterwards, the hydrogel was released from the tube, washed, cut incut into five pieces, and dried as described before. The degree of swelling (Q) was determined by Equation (1).

**Figure 3:**
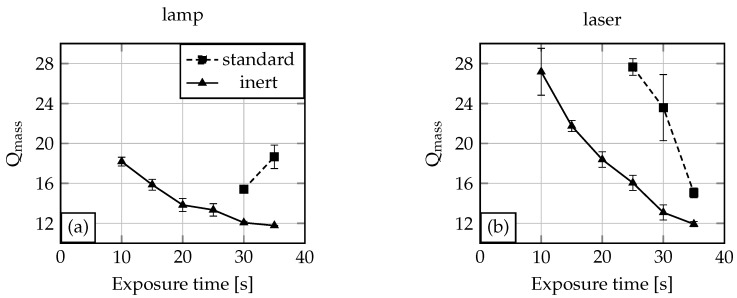
Degree of swelling of Poly-*N*-isopropylacrylamide (PNIPAAm) in water for different exposure times and UV light sources (lamp (**a**), laser (**b**)).

In Figure 3, the results for the exposure time variation experiments are shown. It is demonstrated that for an oxygen-free NMR tube filling inside a glove box, the required time for complete polymerisation (=gelpoint) is 10 s for the lamp (Figure 3a) and also for the laser (Figure 3b). Under standard conditions, 30 s (lamp) or 25 s (laser) exposure time is required to obtain a hydrogel. Shorter exposure times lead to non- or only partially-polymerised solutions. The degree of swelling drops with increasing exposure time. This can be explained by the fact that with longer exposure time the cross-linking density of the gel increases. With an increasing cross-linking density the degree of swelling decreases due to less hydrogen bond formation possibilities [6]. Comparing the inert condition laser and the lamp experiment, the degree of swelling for the laser is overall higher than the degree of swelling for the gels polymerised by the UV lamp. Referring to the light spectra from Figure 2, the UV lamp provides radiation with wavelengths in regions with higher light absorption of the initiator compared to the laser. This yields higher quantum efficiency for the lamp. Therefore, at the same exposure time, more photoinitiator will be excitated by the UV lamp than by the laser. This results in a higher cross-linking density for the gels fabricated with the lamp. For 30 s and 35 s exposure time, the degrees of swelling are nearly the same for both tested light sources. Here the amount of excitated photoinitiator reaches saturation, which leads to comparable cross-linking densities and therefore degrees of swelling.

If the polymerisation solution is filled in the NMR tubes under standard conditions, the polymerisation process needs higher exposure times (see Figure 3). For the experiments conducted with the lamp, the required exposure time for the gel polymerisation was 30 s, and for the laser, 25 s. This and also the rise in the degree of swelling for the 35 s exposure time (lamp) demonstrates the influence of oxygen. Usually polymerisation processes conducted with the lamp should need less or the same exposure time as processes conducted with the laser, due to the better quantum yield. Furthermore, the degree of swelling for a hydrogel polymerised for 35 s should be smaller than the degree of swelling for a 30 s polymerised hydrogel. We explain both phenomena as the influence of undefined oxygen content in the polymerisation solution. This oxygen quenches radicals generated by the UV light and therefore leads to non-conclusive process parameters or hydrogel characteristics.

Preparing hydrogels with comparable characteristics under standard conditions becomes hardly possible due to the low reproducibility of the oxygen content in the polymerisation solution. Comparing the degrees of swelling between the systems with and without oxygen, it can be seen that systems with oxygen show overall a higher degree of swelling than the systems without oxygen. As discussed before, a smaller degree of swelling is a result of higher cross-linking density. Generated photoinitiator radicals due to UV light excitation will be quenched in oxygen-containing systems, and therefore a reduced cross-linking density is the result.

**Figure 4:**
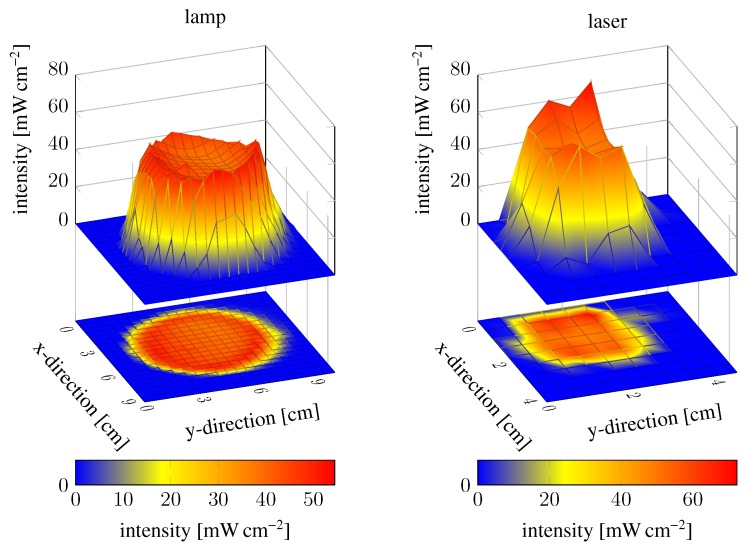
Intensity field distribution of the mercury-vapour lamp and the laser. Measurements were conducted at a distance of 0.5 m for the lamp and 1.5 m for the laser.

The hydrogel polymerisation process depends not only on the oxygen content and exposure time, but also on the radiation intensity of the UV light sources. Higher intensity will speed up the polymerisation process and lower intensity will slow down the process so that shorter or longer exposure times are required, respectively. Therefore, we asked how the intensity distribution of the UV light spots influences the polymerisation. If the UV light intensity distribution is too inhomogeneous, the polymerisation process will also be inhomogeneous, and therefore also the hydrogel characteristics. The field intensity distribution of the mercury-vapour lamp was investigated at 365 nm (highest intensity peak in the bandpass range) at different positions over the whole spot area with a light sensor (100S, Karl Suss, Garching, Germany). The same measurement was conducted for the laser. The results are shown by two surface plots in Figure 4. The intensity at the spot boundaries drops sharply for both sources, so that these areas were avoided during the polymerisation process. Some intense spots could also be measured, but the middle of the spot areas seems to be homogeneous. So these areas were chosen for the polymerisation process.

**Figure 5:**
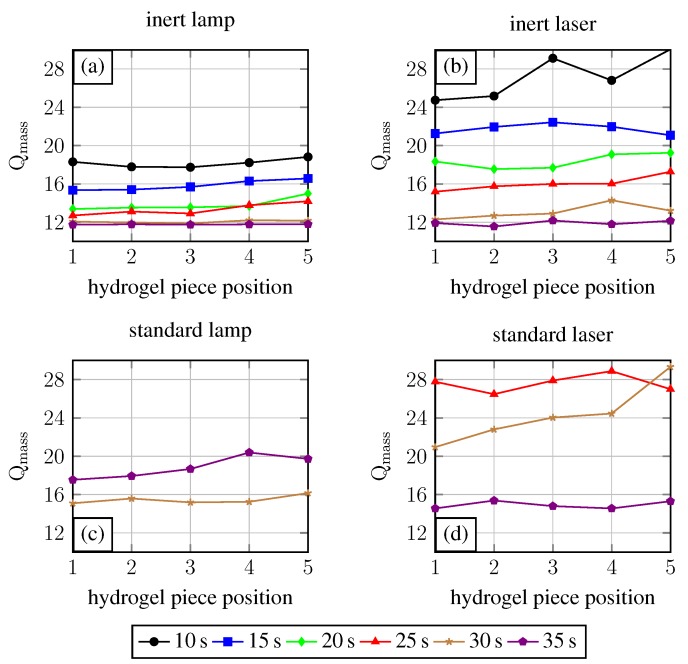
Localised resolution of the polymerisation process. The primary gel were cut incut into five pieces per body and the degrees of swelling were measured.

To exclude the influence of local spot intensities on the polymerisation process, the gels fabricated in the exposure time experiments were cut into five pieces per gel, washed, and dried as described before. In Figure 5, the degree of swelling for the gel pieces is plotted over the position of the gel piece in the original hydrogel body (see Figure 1). If these lines would be straight horizontal lines, this would imply that the fabricated primary hydrogel body has overall comparable characteristics independent from the tube position under the UV light in the polymerisation process. The spot homogeneities of the UV light sources are suitable in the middle, but the boundaries and the intense spots were avoided.

For inert conditions, a nearly-straight horizontal line course is observed. Especially for the lamp with an increasing polymerisation time, this progress becomes obvious. For the laser as the UV light source, the 10 s exposure time shows high alterations in the degree of swelling. Also, the results for the laser look overall more inhomogeneous than the results for the lamp. The reason for the high alteration at the 10 s exposure time with the laser may be the wavelength and therefore quantum yield or the pulsed light. With exposure time elongation, this drawback compared to the lamp will be reduced.

For the systems assembled under standard conditions, higher variations for the degree of swelling are demonstrated in Figure 5. This is explained by a non-uniform oxygen concentration distribution in the systems, which affects the cross-linking density locally in different ways.

We show that the polymerisation process depends on the exposure time and also the oxygen content. By measuring the UV light spots regarding their local intensities and comparing these results with the degree of swelling of hydrogel pieces from the original gel bodies, we demonstrate that the middle of the UV light spots are homogeneous and do not significantly influence the polymerisation process. These findings are important for investigating the reproducibility of the polymerisation process.

### 2.2. Polymerisation Process Reproducibility

**Figure 6:**
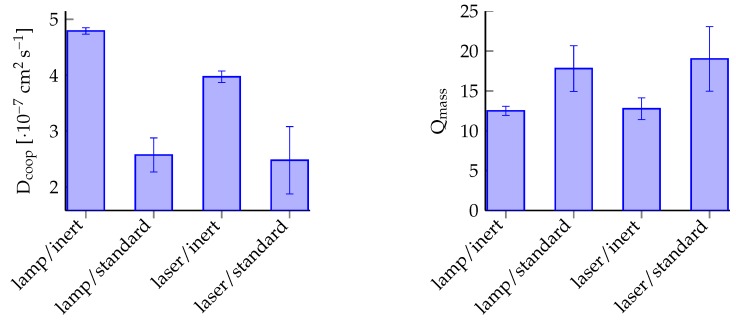
Process reproducibility with the cooperative diffusion coefficient $D_{\mathrm{coop}}$ and the degree of swelling Q as characterisation parameters.

As mentioned in the beginning, the reproducibility of gel characteristics is one of the key conditions for hydrogel applications in microsystem engineering. To investigate this, 3 NMR tubes for each experimental condition (inert or standard with lamp or laser respectively) were exposed to UV light for 30 s because at this exposure time an overall hydrogel formation could be observed (see Figure 3). Afterwards DLS measurements supply the cooperative diffusion coefficient $D_{\mathrm{coop}}$ for each hydrogel. The diffusion coefficients and the degree of swelling were compared (see Figure 6). For the degree of swelling measurements, gel bodies were cut into pieces and degrees of swelling were averaged.

For the evaluation of the reproducibility, the percentage on the mean for each experimental condition and characterisation parameter were used. The polymerisation process under these experimental conditions are for systems under inert conditions—the value is always significantly smaller than for systems under standard conditions (see Table 1). Also, the value for polymerisation under inert conditions with the mercury-vapour lamp is smaller for polymerisation with the laser. Therefore we got the highest reproducibility for an oxygen-free polymerisation with a UV lamp as radiation source.

Furthermore, inert polymerisation with the lamp yields the highest hydrogel cross-linking densities. With increasing cross-linking density, the degree of swelling decreases and the cooperative diffusion coefficient increases [23]. Following this interpretation, the polymerisation (independent of oxygen content) with the laser causes a smaller degree of cross-linking than the mercury-vapour lamp, as shown in Figure 6. Therefore a polymerisation process initiated by the laser used herein is favorable for hydrogels with high degrees of swelling.

### 2.3. Improved Hydrogel Microstructuring

To apply our previous findings, we performed photostructuring experiments to show the improvement in hydrogel photopatterning in the micrometer range. Following our previous findings we decided to use the mercury-vapour lamp as UV light source.

**Figure 7:**
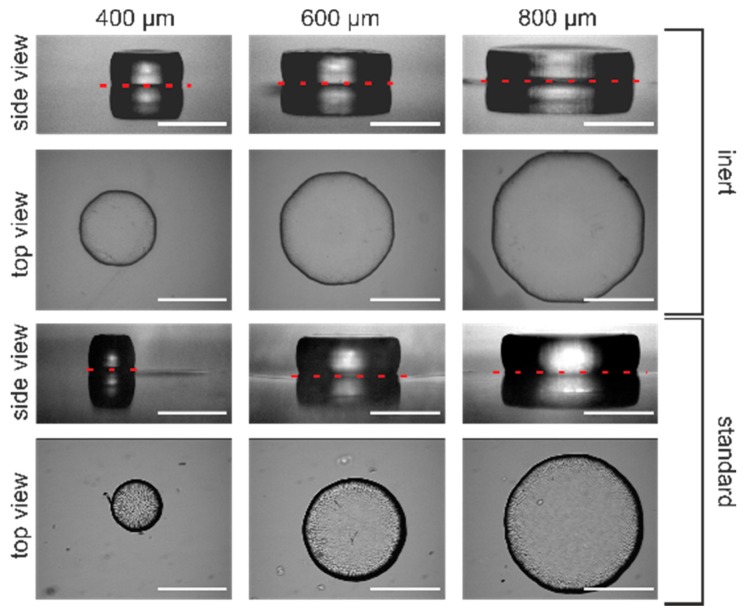
Results from polymerisation processes under different conditions. Hydrogels were covalently cross-linked to the glass surface during the polymerisation process. Therefore, a reflection of the gels in the side views is observable with the plane of reflection indicated by the red dashed lines (white bar = 400 μm).

Figure 7 displays the results from hydrogel microstructuring experiments for different mask apertures ranging from 400 μm to 800 μm. The hydrogel diameter for glove box-assembled systems (inert conditions) are close to the mask aperture (see Figure 7). For the systems assembled under standard conditions, the hydrogel diameter is always smaller than the size of the mask aperture. Interestingly, in the case of the 400 μm aperture size in standard conditions, the resulting gel has a diameter of only 270 μm. The reason for this sparse result is that the polymerisation process gets quenched by oxygen. This becomes more critical with reduction of the photomask aperture and therefore hydrogel downscaling. With a reduction of photomask aperture, scattering effects at the mask boundaries become intensified, hence less light excites photoinitiator molecules. If there are excited photoinitiator molecules forming radicals for polymerisation, these radicals will be quickly quenched by oxygen, resulting in smaller hydrogel diameters than the mask aperture prompts.

For microsystems applications, these results demonstrate in the best way the necessity to conduct the polymerisation process under inert conditions. If hydrogel downscaling becomes essential, e.g., for high integration densities of hydrogel actuators, the polymerisation has to be conducted under inert conditions to guarantee pattern fidelity. Consequently, working under inert conditions not only improves the reproducibility of the polymerisation process but also the resolution of the photopatterning process.

## 3. Conclusions

In this paper we discussed the polymerisation procedure for PNIPAAm hydrogels under different conditions. We investigated the influence of different oxygen content in the polymerisation setup and also the influence of different UV radiation sources, where we introduce a pulsed laser as UV source (51 kHz, *λ* = 355 nm). We found that assembling the polymerisation setup under inert gas conditions improves the reproducibility of hydrogel characteristics and therefore the hydrogel itself. By comparing the percentage of the standard deviations (standard and inert conditions) for both UV sources, the height of the improvement was 10 times (lamp) or 12 times (laser) for the diffusion coefficient, and 4 times (lamp) or 2 times (laser) for the degree of swelling, respectively. From an engineer point of view, the reproducibility of hydrogel characteristics (e.g., size, degree of swelling, cooperative diffusion coefficient) is one of the key features to use hydrogels in microsystem engineering. We could also demonstrate an improvement in the photopatterning process by using these findings. These result in better downscaling behaviour, and therefore in a resolution enhancement, which solves the limitation of decreasing pattern size. With the results shown here, we recommend a polymerisation setup assembled under inert gas conditions. Results at this optimisation state indicate the advantages of continous wide-band UV lamp with wavelengths in the range of 300 nm to 400 nm.

## 4. Outlook

For further improvement of the polymerisation process, we consider some process parameters. We believe that with a laser optimisation time mode (pulse frequency), another wavelength, or another photoinitiator whose absorption spectra fits better to the spectra of the laser used herein a further optimisation of the polymerisation process should be possible.

## 5. Experimental Section

### 5.1. Chemicals

*N*-Isopropylacrylamide (NIPAAm), *N,N${}^{\prime }$*-Methylene-bis-acrylamide (BIS), photoinitiator Irgacure^®^ 2959, and 3-(trimethoxysilyl) propyl methacrylate were purchased from Sigma-Aldrich (Sigma-Aldrich, St. Louis, USA). NIPAAm was recrystallised from n-hexane (VWR International GmbH, Darmstadt, Germany), and other chemicals were used as received. All experiments were conducted in deionised water with an electrical conductivity of 0.056 μS·cm${}^{-1}$ (Barnstead GENPURE PRO, Thermo Scientific, Langenselbold, Germany), generated by ion exchange.

### 5.2. Preparation of Polymerisation Solution A

Polymerisation solution was prepared by dissolving 2.122 g NIPAAm and 0.058 g BIS in 15 mL water. The solution was flushed by bubbling argon through the solution for 1 h to remove oxygen, afterwards sealed with a septum, and brought into a glove box. 0.042 g photoinitiator was added, and the solution was thoroughly mixed.

### 5.3. Polymerisation Process Characterisation

For oxygen-free polymerisation systems preparation, solution A (1 mL) was filtered with a syringe filter (pore size 0.45 μm, VWR International GmbH, Darmstadt, Germany), and filled in NMR tubes (Deutero GmbH, Kastellaun, Germany) inside of the argon filled glove box (= inert conditions). The oxygen content in the glove box was less than 0.5 ppm. Otherwise, the solution was filtered in the glove box (mBraun, LAB Star, Garching, Germany) and subsequently filled in NMR tubes outside of the glove box (= standard conditions). Exposure times for the photopolymerisation process ranged from 10 s to 35 s in 5 s steps.

To avoid inhomogeneities in the polymer due to tube thickness, the NMR tubes were rotated during exposure time. For rotation of the NMR tube during the polymerisation process, a rotating system was developed (see Figure 1), forced by a coupling between an electrically-driven motor and the NMR tube realised by a metal chain and two gears. The motor rotation is brought to the NMR tube by the chain and a further gear. Here the NMR tube acts as a second shaft with two bearings. To exclude thermal effects, the reaction mixture is cooled to 0 °C with an ice bath.

UV light sources were a mercury-vapour lamp (1000 W Hg(Xe), L.O.T.-Oriel Group Europe, Darmstadt, Germany) (spectra in Figure 2) or a laser with a wavelength of *λ* = 355 nm and a pulse frequency of 51 kHz (AVIA 355-5, Coherent, Dieburg, Germany).

### 5.4. Hydrogel Characteristics Determination—Degree of Swelling

Weight measurements were done with a precision balance (BP 210 S, Sartorius, Goettingen, Germany). For mass measurement, hydrogels were cut incut into pieces and degrees of swelling were determined following Equation (1). The following three-step cycle was repeated five times. First, the hydrogels were swollen in water for 24 h (step 1). After the incubation, the hydrogels were deswollen at 60 °C for 2 h in a convection oven (Venti-Line, VWR International GmbH, Darmstadt, Germany) (step 2). Following the deswelling, the water was exchanged to fresh water (step 3), and incubation was again carried out for 24 h (step 1). ThisThese cycles, including swelling and deswelling steps, are necessary to wash out non-polymerised parts. After the fifth cycle, hydrogels were again deswollen, water was decanted, and the hydrogels were dried for 24 h at 60 °C in a convection oven. The lingering water content was removed by drying the hydrogels for 60 h at 60 °C in a vacuum oven (Heraeus, Thermo Scientific, Langenselbold, Germany). Hydrogels were weighed and consecutively incubated for 72 h in water to calculate the degree of swelling afterwards.

(1) $$Q_{\mathrm{mass}}=\frac{{\mathrm{mass}}_{\mathrm{swollen}}}{{\mathrm{mass}}_{\mathrm{dried}}}$$

The characteristic of choice for evaluation of the reproducibility was the standard deviation (SD). For a better comparison, the percentage of the standard deviation on the mean was calculated.

### 5.5. Determination of Cooperative Diffusion Coefficient

The cooperative diffusion coefficient was measured by dynamic light scattering (DLS, also known as photon correlation spectroscopy). With these measurements, the diffusion coefficient was determined for each hydrogel prepared in a NMR tube [24,25].

DLS measurements were carried on an ALV-5000 compact goniometer system (ALV, Langen, Germany) equipped with a helium-neon laser (*λ* = 632.8 nm) and coupled with an ALV photon correlator. Samples were measured at a scattering angle of *θ* = 90${}^{{}^{\circ}}$. A toluene bath was used to match the refractive index and control the temperature at 25 °C. The time-averaged scattering intensities $<I{>}_{T}$ and the time-averaged intensity correlation functions (ICF, $g_{T}^{\left(2\right)}\left(q,\tau \right)-1$) were determined at 50 different sample positions selected by randomly rotaterandomly rotating the cuvette before each run. The time for each run was 30 s. From the ICFs measured at each position, apparent diffusion coefficients $D_{A}$ were estimated according to Equation (2).

(2) $$D_{A}=-\frac{1}{2q^{2}}\lim_{\tau \to 0}\frac{\delta }{\delta t}\ln\left({g_{T}}^{\left(2\right)}\left(q,\tau \right)-1\right)$$

here, q = (4Πn/$\lambda_{0}$) sin(*θ*/2) is the scattering vector, with *θ* being the scattering angle, $\lambda_{0}$ the wavelength of the incident light in a vacuum, and n the refractive index of the medium. For different sample positions, different values of D${}_{A}$ and the local scattering intensities $<I{>}_{T}$ were obtained. The relationship between $D_{A}$ and the cooperative diffusion coefficient $D_{\mathrm{coop}}$ is given by Equation (3).

(3) $$\frac{<I{>}_{T}}{D_{A}}=\frac{2}{D_{\mathrm{coop}}}<I{>}_{T}-\frac{<I_{F}{>}_{T}}{D_{\mathrm{coop}}}$$

Plotting $<I{>}_{T}/D_{A}$ *versus* $<I{>}_{T}$, the data formed essentially a straight line, from whose slope and intercept the fluctuating component of the scattering intensity, $<I_{F}{>}_{T}$, as well as $D_{\mathrm{coop}}$, were obtained.

### 5.6. Hydrogel Microstructuring

Photostructuring experiments were conducted under inert gas conditions or atmosphere. First, cover slips (VWR International GmbH, Darmstadt, Germany) were cleaned with acetone, isopropanol, as well as water and dried under nitrogen stream. The glass surface was activated by oxygen plasma for 2 min at 50 W (Hochvakuum Dresden GmbH, Dreva Clean 450, Dresden, Germany) for subsequent silanisation. The activated glass slides were immersed in a 2% *v*/*v* ethanolic solution with 3-(trimethoxysilyl) propyl methacrylate for 2 h to get a methacrylic surface modification of the slides for covalent bonding of the polymerised hydrogels to the glass surface. Afterwards, the slides were rinsed with water and dried under a stream of nitrogen. Following that, a polymerisation chamber was assembled and the polymerisation solution was added. This assembling procedure was conducted inside (inert conditions) or outside (standard conditions) of the glove box, respectively. Finally, the polymerisation was directly carried out for 60 s (inert conditions) or 90 s (standard conditions) under a mercury-vapour lamp through a polymer film mask.

## Figures and Tables

**Table 1:** Hydrogel characteristics cooperative diffusion coefficient $D_{\mathrm{coop}}$ and degree of swelling $Q_{\mathrm{mass}}$ at different polymerisation conditions (standard or inert, lamp or laser).

Condition	$D_{\mathrm{coop}}$	${\mathrm{SD}}_{D_{\mathrm{coop}}}$		$Q_{\mathrm{mass}}$	${\mathrm{SD}}_{Q}$	
Unit	(cm${}^{-2}$·s${}^{-1}$)	(cm${}^{-2}$·s${}^{-1}$)	%	(-)	(-)	%
lamp, inert	4.79 × 10^−7^	5.64 × 10^−9^	1.18	12.50	0.58	4.61
lamp, standard	2.57 × 10^−7^	3.05 × 10^−8^	11.84	17.81	2.87	16.11
laser, inert	3.97 × 10^−7^	1.04 × 10^−8^	2.61	12.76	1.37	10.70
laser, standard	2.48 × 10^−7^	6.01 × 10^−8^	24.23	19.02	4.06	21.33
